# First report of field evolved resistance to agrochemicals in dengue mosquito, *Aedes albopictus *(Diptera: Culicidae), from Pakistan

**DOI:** 10.1186/1756-3305-4-146

**Published:** 2011-07-22

**Authors:** Hafiz Azhar Ali Khan, Waseem Akram, Khurram Shehzad, Essam A Shaalan

**Affiliations:** 1 Department of Entomology, University College of Agriculture, Bahauddin Zakariya University, Multan, Pakistan; 2 Department of Agri-Entomology, University of Agriculture, Faisalabad, Pakistan; 3 Department of Biology, Islamia University of Bahawalpur, Pakistan; 4 Department of Zoology, South Valley University, Aswan, Egypt

## Abstract

**Background:**

Agrochemicals have been widely used in Pakistan for several years. This exposes mosquito populations, particularly those present around agricultural settings, to an intense selection pressure for insecticide resistance. The aim of the present study was to investigate the toxicity of representative agrochemicals against various populations of *Aedes albopictus *(Skuse) collected from three different regions from 2008-2010.

**Results:**

For organophosphates and pyrethroids, the resistance ratios compared with susceptible Lab-PK were in the range of 157-266 fold for chlorpyrifos, 24-52 fold for profenofos, 41-71 fold for triazofos, and 15-26 fold for cypermethrin, 15-53 fold for deltamethrin and 21-58 fold for lambdacyhalothrin. The resistance ratios for carbamates and new insecticides were in the range of 13-22 fold for methomyl, 24-30 fold for thiodicarb, and 41-101 fold for indoxacarb, 14-27 fold for emamectin benzoate and 23-50 fold for spinosad. Pair wise comparisons of the log LC_50s _of insecticides revealed correlation among several insecticides, suggesting a possible cross resistance mechanism. Moreover, resistance remained stable across 3 years, suggesting field selection for general fitness had also taken place for various populations of *Ae. albopictus*.

**Conclusion:**

Moderate to high level of resistance to agrochemicals in Pakistani field populations of *Ae. albopictus *is reported here first time. The geographic extent of resistance is unknown but, if widespread, may lead to problems in future vector control.

## Background

Dengue fever and dengue hemorrhagic fever (DF/DHF) are vector borne diseases of public health concerns in tropical and subtropical parts of the world [1], affecting millions of people annually [2]. The incidence of DF and DHF has increased cyclically in Pakistan since the first recognized outbreak in 1994 with *Ae. albopictus *(Skuse) as the core mosquito vector in this respect [3]. Currently, controlling this vector with insecticidal habitat spraying remains an important option to minimize the incidence of dengue fever [4], resulting in resurgence and development of insecticidal resistance.

Insecticide resistance has become a limiting factor in the use of these compounds in chemical control of many insect pests. The exploration of more efficient toxic chemicals and other control tactics are necessary with the increasing world population and preservation of species diversity [5]. Frequent use of chemicals, such as pesticides, coupled with monoculture crops on a large scale, has generated pesticide resistance in insect pests, resurgence and difficulties in pest management [6]. By 2007, intensive use of pesticides had resulted in at least 553 arthropod species resistant to one or more classes of insecticides (organochlorines, organophosphates, carbamates and pyrethroids) [7]. Of these, 60 percent are agricultural pests and the remaining 40 percent are pests of medical importance [8]. Resistance in medical pests or disease vectors is a serious threat to the control of vector-borne diseases, owing to the fact of insecticide-based strategies such as insecticide treated nets, indoor residual spraying, insecticide treatment of breeding habitats and also because of agricultural practices [9].

Various disease vectors are present in agro-ecozones and are therefore likely to be exposed to chemicals used to control agricultural pests. Despite the lack of concrete evidence, the massive use of agrochemicals has been considered as a key factor contributing to the emergence of vector resistance to insecticides [10]. Insecticide resistance in disease vectors due to the selection pressure from agrochemicals has been reported from different parts of the world [9,11-15], however, no such reports have so far been reported from Pakistan.

Crop losses caused by insect pest in Pakistan are upto 56%, and 20-40% of these losses are in cotton, *Gossypium hirsutum *L. [16]. As a result, agrochemicals with broad toxicity to target pests and non target organisms are being widely used in cotton insect pest management. The overuse of chemicals can lead to the phenomenon of insecticide resistance both in target and non target organisms. In the current study, we were interested to establish whether *Ae. albopictus*, present in cotton cultivated fields, had developed resistance to agrochemicals (organophosphates, carbamates, pyrethroids and newer compounds). These chemicals are commonly used for the control of cotton insect pests in Punjab province, Pakistan [17]. We were also interested in investigating whether resistance to different insecticides was increasing or remained the same from 2008-2010. The present paper reports the first known occurrence of high level resistance to agrochemicals in *Ae. albopictus*. The data from such studies are expected to help in future management strategies so that the development of resistance is delayed to a maximum in *Ae. albopictus *under field conditions of Pakistan.

## Materials and methods

### Mosquitoes

We collected natural populations of *Ae. albopictus *from upper Punjab, Pakistan (Lahore, Sargodha and Faisalabad districts) from 2008-2010. The growers usually undertake more insecticides on cotton than any other crop [17]. We therefore collected *Ae. albopictus *populations from cotton fields as there were higher chances of evolution of resistance on cotton than other crops. The collection sites within the districts were kept constant across three years. Moreover, a group of *Ae. Albopictus *collected in a date from a determinate place was considered as a population. The samples of larvae and pupae from each district were colonized under laboratory conditions at 27 ± 1°C and 65 - 70% RH. Larvae were fed on fish food (TetraMin^®^). Adults were kept in plastic cages (30 × 40 × 40) where males were provided cotton wicks soaked with 20% sucrose solution and females were fed on blood of white rats thrice a week [4]. Fourth instar larvae of the F1 progeny were reared for bioassays. However, some bioassays were performed on the F2 generation due to insufficient numbers of F1 progeny. The laboratory susceptible strain of *Ae. albopictus *was collected in 2005 from mountainous areas of Islamabad with zero or very low chemical use and it was designated as Lab-PK. This population was reared in the laboratory for >40 generations without exposure to insecticides. The Lab-PK population showed lowest LC_50 _values for all the tested insecticides, and hence was used as a reference strain to calculate resistance ratios.

### Insecticides

Commercial formulations of different insecticides used for bioassays consisted of chlorpyrifos (Lorsban 40 EC, Dow Agro Sciences, United Kingdom), profenofos (Curacron 50 EC, Syngenta Crop Protection, Switzerland), triazofos (Hostathion 40 EC, Bayer Crop Science), cypermethrin (Arrivo 10 EC, FMC, Philadelphia; PA), deltamethrin (Decis Super 10.5 EC, Bayer Crop Science, Multan, Pakistan), lambdacyhalothrin (Karate EC Syngenta Crop Protection Switzerland ), methomyl (Lannate LV 239 g [AI]/liter, DuPont, Pakistan), thiodicarb (Larvin SC 375 g [AI]/liter, Bayer Crop Science, Multan, Pakistan), indoxacarb (Steward 15SC, DuPont, Pakistan), spinosad (Tracer 24SC, Dow Agro Sciences, UK) and emamectin benzoate (Proclaim 1.9 EC, Syngenta, UK)

### Bioassays

Bioassays were performed as described previously [18] using acetone solution of insecticides. One milliliter of appropriate insecticide solution was dispensed with a pipette above the water surface in each glass beaker containing 99 ml of distilled water. Each insecticide was tested within a range of seven to eight concentrations to determine LC_50 _value, including controls, and each concentration was replicated at least four times. Ten 4^th ^instar larvae were placed in the glass beaker in each replication and the total number of larvae tested per concentration was 40. The bioassays were kept at a temperature of 27 ± 1°C, 65% RH and a photoperiod of 14L: 10D hours. Mortality was recorded after 24 hours [18], except for spinosad, which was assayed after 48 hours due to the slower acting nature of this insecticide. Larvae were considered dead if they could not be induced to move when probed with a probe.

### Stability of resistance

A decline or increase in resistance to the tested insecticides in field populations from 1 year to the next was measured by calculating R values i.e., respond per month. The R values were estimated as below:

Where '*n' *is the number of months (6 months) after which a second population was collected from the same field. Decline or increase in resistance is presented in - and/or + values of *R *[16].

### Data analysis

Mortality data, where necessary, were corrected by Abbott's formula [19]. Data were analyzed using probit analysis based on Finney (1971) [20] to determine the LC_50 _values and their 95% fiducial limits (FLs) using MINITAB 15 statistical software [21]. Due to the inherent variability of bioassays, pair wise comparisons of LC_50 _values were made, and if 95% FLs of two treatments did not overlap at 1% level of significance, they were considered significant [22]. Resistance ratios (RRs) were calculated by dividing the LC_50 _values of field populations with LC_50 _of susceptible Lab-PK. To determine cross resistance among the tested insecticides, pair wise correlation coefficients (*r*) of log LC_50 _values of the field populations were also calculated. The slopes of regression lines were compared using *t*-test in Statistix 8.1 [23].

To determine insecticide resistance, the level of insecticide resistance was scaled by using resistance ratios (RRs) in terms of widely accepted values as follows: susceptibility (RR = 1), low resistance (RR = 2-10), moderate resistance (RR = 11-30), high resistance (RR = 31-100) and a very high resistance (RR > 100) [24].

## Results

### Toxicity of insecticides to susceptible population

The results obtained from the bioassays with the Lab-PK population (Table 1, 2) revealed that chlorpyrifos was significantly more toxic (non overlapping of 95% FL; P < 0.01) than the insecticides tested viz., profenofos, triazofos, cypermethrin, deltamethrin, lambdacyhalothrin, methomyl, thiodicarb, indoxacarb, emamectin benzoate and spinosad. Emamectin benzoate was the least effective compound than the other insecticides tested. The slopes of regression lines of all the insecticides were similar (P > 0.05).

**Table 1:** Toxicity of organophosphates and pyrethroids against field populations of *Ae. albopictus*

Insecticide	Location	Time	LC_50 _(95% FL) (µg mL^-1^)	Slope ± SE	χ^2^	df	P	RR*	DR**	n***
**Chlorpyrifos**	Lab-PK		0.009 (0.002-0.013)	2.25 ± 0.31	0.69	5	0.98	1	_	280
	Lahore	Mar. 2009	1.92 (1.27-4.95)	1.04 ± 0.25	5.73	6	0.46	156.6	_	320
		Sep. 2009	2.61 (1.35-13.21)	0.59 ± 0.26	5.84	6	0.44	247.8	0.222	320
		Mar. 2010	2.88 (1.27-15.44)	0.56 ± 0.25	3.95	6	0.68	242.2	0.030	320
		Sep. 2010	3.36 (1.33-8.55)	0.67 ± 0.26	2.65	6	0.85	240	0.041	320
	Faisalabad	Sep. 2008	2.08 (1.26-7.31)	0.66 ± 0.30	3.34	6	0.77	224.4	_	320
		Mar. 2009	2.40 (1.39-18.57)	0.59 ± 0.27	5.51	6	0.48	266	0.010	320
		Sep. 2009	1.71 (1.19-3.48)	0.93 ± 0.27	5.76	6	0.45	190	-0.014	320
		Mar. 2010	2.00 (1.32-5.15)	0.84 ± 0.28	6.11	6	0.41	222	-0.003	320
		Sep. 2010	2.12 (1.37-6.32)	0.81 ± 0.29	6.21	6	0.40	235.6	0.001	320
	Sargodha	Sep. 2008	1.74 (1.15-4.29)	0.77 ± 0.25	6.51	6	0.37	193	_	320
		Mar. 2009	1.89 (1.25-4.77)	0.81 ± 0.27	9.40	6	0.15	210	0.006	320
		Sep. 2009	1.57 (1.15-2.76)	1.09 ± 0.27	11.3	6	0.08	174	-0.007	320
		Mar. 2010	1.48 (1.16-2.23)	1.57 ± 0.35	9.45	6	0.15	164	-0.012	320
		Sep. 2010	1.69 (1.24-3.00)	1.22 ± 0.32	10.3	6	0.11	187.8	-0.002	320
**Profenofos**	Lab-PK		0.02 (0.015-0.04)	2.41 ± 0.27	2.44	6	0.88	1	_	320
	Lahore	Sep. 2008	0.56 (0.43-0.77)	1.25 ± 0.22	9.34	6	0.16	28	_	320
		Mar. 2009	0.70 (0.53-1.04)	1.09 ± 0.31	5.24	5	0.39	35	0.016	280
		Sep. 2009	0.73 (0.55-1.07)	1.10 ± 0.40	7.49	6	0.28	36.5	0.019	320
		Mar. 2010	0.79 (0.59-1.23)	1.05 ± 0.23	5.90	6	0.43	39.5	0.025	320
		Sep. 2010	0.82 (0.52-2.26)	0.62 ± 0.23	0.53	5	0.99	41	0.028	280
	Faisalabad	Sep. 2008	0.80 (0.62-1.15)	1.17 ± 0.33	11.6	6	0.07	40	_	320
		Mar. 2009	0.82 (0.64-1.18)	1.19 ± 0.24	3.90	6	0.69	41	0.001	320
		Sep. 2009	0.98 (0.75-1.48)	1.11 ± 0.40	7.38	6	0.29	49	0.015	320
		Mar. 2010	1.03 (0.77-1.62)	1.03 ± 0.37	8.62	6	0.17	51.5	0.018	320
		Sep. 2010	0.71 (0.56-0.96)	1.31 ± 0.23	9.78	6	0.13	35.5	-0.009	320
	Sargodha	Sep. 2008	0.49 (0.35-0.72)	0.97 ± 0.20	1.95	5	0.86	24	_	280
		Mar. 2009	0.54 (0.42-0.73)	1.44 ± 0.23	9.09	6	0.17	27	0.007	320
		Sep. 2009	0.68 (0.52-0.94)	1.23 ± 0.32	6.44	6	0.38	34	0.024	320
		Mar. 2010	0.52 (0.35-0.84)	0.91 ± 0.25	2.08	5	0.84	26	0.004	280
		Sep. 2010	0.55 (0.39-0.74)	1.23 ± 0.25	8.09	6	0.17	27.5	0.008	320
**Triazofos**	Lab-PK		0.036 (0.02-0.06)	2.39 ± 1.29	8.06	6	0.23	1	_	320
	Lahore	Sep. 2008	1.80 (1.18-4.71)	0.75 ± 0.20	8.18	6	0.22	50	_	320
		Mar. 2009	1.94 (1.28-4.99)	0.82 ± 0.19	11.8	6	0.07	53.9	0.005	320
		Sep. 2009	1.49 (1.11-2.44)	1.16 ± 0.27	7.82	6	0.25	41.4	-0.014	320
		Mar. 2010	1.62 (1.22-2.70)	1.36 ± 0.33	8.20	6	0.23	45	-0.008	320
		Sep. 2010	1.80 (1.28-3.50)	1.12 ± 0.31	7.57	6	0.27	50	0	320
	Faisalabad	Sep. 2008	2.26 (1.31-12.26)	0.58 ± 0.16	5.89	5	0.32	62.8	_	280
		Mar. 2009	2.00 (1.29-6.08)	0.79 ± 0.29	10.0	5	0.08	55.6	-0.009	280
		Sep. 2009	2.26 (1.38-11.23)	0.70 ± 0.29	6.29	5	0.28	62.8	0	280
		Mar. 2010	2.26 (1.42-8.69)	0.81 ± 0.32	4.33	5	0.50	62.8	0	280
	Sargodha	Sep. 2008	2.57 (0.55-4.51)	0.64 ± 0.29	4.73	6	0.58	71.4	_	320
		Mar. 2009	1.96 (0.88-3.01)	0.81 ± 0.27	8.49	6	0.21	54.5	-0.019	320
		Sep. 2009	1.72 (0.99-2.50)	1.04 ± 0.29	11.3	6	0.08	47.8	-0.029	320
		Mar. 2010	1.74 (0.92-2.61)	0.86 ± 0.40	5.63	6	0.47	48.3	-0.028	320
		Sep. 2010	2.18 (0.89-3.47)	0.87 ± 0.31	3.01	6	0.81	60.6	-0.012	320
**Cypermethrin**	Lab-PK		0.04 (0.02-0.09)	2.41 ± 0.45	5.96	6	0.43	1	_	320
	Lahore	Sep. 2008	0.86 (0.66-1.29)	1.11 ± 0.23	8.62	6	0.20	21.5	_	320
		Mar. 2009	0.71 (0.57-0.95)	1.40 ± 0.35	11.6	6	0.72	17.8	-0.014	320
		Sep. 2009	0.63 (0.52-0.85)	1.46 ± 0.22	10.4	6	0.11	15.8	-0.023	320
		Mar. 2010	0.89 (0.69-1.30)	1.16 ± 0.24	11.3	6	0.08	22.3	0.003	320
		Sep. 2010	0.83 (0.64-1.15)	1.24 ± 0.42	6.43	6	0.38	20.8	-0.003	320
	Faisalabad	Sep. 2008	1.02 (0.78-1.54)	1.11 ± 0.37	9.79	6	0.13	25.5	_	320
		Mar. 2009	0.78 (0.55-1.00)	1.19 ± 0.43	6.56	6	0.36	19.5	-0.019	320
		Sep. 2009	0.94 (0.54-1.34)	0.82 ± 0.22	4.21	6	0.65	23.5	-0.006	320
		Mar. 2010	0.94 (0.60-1.27)	1.00 ± 0.30	6.29	6	0.39	23.5	-0.006	320
		Sep. 2010	0.87 (0.53-1.21)	0.87 ± 0.23	4.03	5	0.55	21.8	-0.012	280
	Sargodha	Sep. 2008	0.68 (0.52-0.83)	1.49 ± 0.41	9.15	6	0.17	17	_	320
		Mar. 2009	0.58 (0.46-0.70)	1.55 ± 0.24	11.9	6	0.06	14.5	-0.012	320
		Sep. 2009	0.69 (0.50-0.87)	1.26 ± 0.44	7.49	6	0.28	17.3	0.001	320
		Mar. 2010	0.70 (0.55-0.86)	1.57 ± 0.52	8.37	6	0.21	17.5	0.002	320
		Sep. 2010	0.66 (0.49-0.83)	1.35 ± 0.37	10.5	6	0.11	16.5	-0.002	320
**Deltamethrin**	Lab-PK		0.028 (0.02-0.04)	2.29 ± 0.24	3.39	6	0.76	1	_	320
	Lahore	Sep. 2008	1.06 (0.74-1.34)	1.18 ± 0.18	4.08	6	0.67	37.9	_	320
		Mar. 2009	1.22 (0.76-1.68)	0.98 ± 0.25	4.34	6	0.63	43.6	0.010	320
		Sep. 2009	0.92 (0.61-1.23)	1.02 ± 0.23	4.49	6	0.61	32.9	-0.010	320
		Mar. 2010	0.41 (0.262-5.61)	0.82 ± 0.13	0.55	4	0.97	14.6	-0.069	240
		Sep. 2010	1.15 (0.57-1.74)	0.72 ± 0.16	2.25	5	0.81	41.1	0.006	280
	Faisalabad	Sep. 2008	1.48 (0.82-2.14)	0.88 ± 0.21	4.12	6	0.66	52.8	_	320
		Mar. 2009	1.35 (0.77-1.92)	0.92 ± 0.36	1.57	5	0.91	48.2	-0.007	280
		Sep. 2009	1.16 (0.72-1.60)	0.95 ± 0.21	3.78	6	0.71	41.4	-0.018	320
		Mar. 2010	1.27 (0.70-1.83)	0.82 ± 0.40	3.81	6	0.70	45.4	-0.011	320
		Sep. 2010	1.31 (0.77-1.71)	1.04 ± 0.38	8.61	6	0.17	46.8	-0.009	320
	Sargodha	Sep. 2008	0.70 (0.51-0.88)	1.33 ± 0.52	6.10	6	0.41	25	_	320
		Mar. 2009	0.88 (0.63-1.13)	1.21 ± 0.28	5.52	6	0.48	31.4	0.017	320
		Sep. 2009	0.63 (0.49-0.77)	1.66 ± 0.34	11.9	6	0.06	22.5	-0.008	320
		Mar. 2010	0.68 (0.44-0.99)	0.99 ± 0.42	5.65	6	0.46	24.3	-0.002	320
		Sep. 2010	0.68 (0.51-0.83)	1.44 ± 0.36	9.20	6	0.16	24.3	-0.002	320
**Lambdacyh-alothrin**	Lab-PK		0.02 (0.014-0.033)	2.32 ± 0.51	3.56	6	0.74	1	_	320
	Lahore	Sep. 2008	0.91 (0.59-1.22)	1.00 ± 0.16	9.41	5	0.09	45.5	_	280
		Mar. 2009	1.16 (0.72-1.60)	0.97 ± 0.24	8.61	5	0.13	58	0.018	280
		Sep. 2009	0.91 (0.61-1.20)	1.10 ± 0.26	9.22	5	0.10	45.5	0	280
		Mar. 2010	0.90 (0.56-1.23)	0.92 ± 0.28	8.24	5	0.14	45	-0.001	280
		Sep. 2010	1.15 (0.63-1.66)	0.81 ± 0.18	6.58	5	0.25	57.5	0.017	280
	Faisalabad	Sep. 2008	0.84 (0.57-1.11)	1.05 ± 0.20	9.28	6	0.16	42	_	320
		Mar. 2009	0.65 (0.44-0.87)	1.10 ± 0.31	9.71	6	0.14	33	-0.018	320
		Sep. 2009	0.83 (0.61-1.05)	1.28 ± 0.44	12.0	6	0.06	41.5	-0.001	320
		Mar. 2010	0.81 (0.59-1.03)	1.27 ± 0.36	8.85	6	0.18	40.5	-0.003	320
		Sep. 2010	0.78 (0.52-1.04)	1.03 ± 0.26	8.41	6	0.21	39	-0.005	320
	Sargodha	Sep. 2008	0.42 (0.30-0.54)	1.42 ± 0.61	10.7	6	0.09	21	_	320
		Mar. 2009	0.59 (0.46-0.72)	1.62 ± 0.46	10.1	6	0.12	29.5	0.025	320
		Sep. 2009	0.64 (0.49-0.80)	1.56 ± 0.44	10.4	6	0.11	32	0.030	320
		Mar. 2010	0.55 (0.43-0.68)	1.56 ± 0.48	8.10	6	0.23	27.5	0.020	320
		Sep. 2010	0.65 (0.45-0.86)	1.09 ± 0.26	7.05	6	0.32	32.5	0.032	320

*Resistance ratio = LC_50 _field population/LC_50 _of susceptible strain

**Rate of increase or decrease in resistance, *** Number of larvae tested in a bioassay

**Table 2:** Toxicity of carbamates and new insecticides against field populations of *Ae. albopictus*

Insecticide	Location	Time	LC_50 _(95% FL) (µg mL^-1^)	Slope ± SE	χ^2^	df	P	RR*	DR**	n***
**Methomyl**	Lab-PK		0.06 (0.03-0.17)	2.56 ± 033	9.99	6	0.12	1	_	320
	Lahore	Sep. 2008	1.31 (0.71-1.91)	0.81 ± 0.40	3.13	6	0.79	21.8	_	320
		Mar. 2009	1.11 (0.63-1.51)	0.84 ± 0.26	4.61	6	0.59	18.5	-0.012	320
		Sep. 2009	0.95 (0.66-1.24)	1.14 ± 0.23	10.5	6	0.10	15.8	-0.023	320
		Mar. 2010	0.92 (0.70-1.14)	1.46 ± 0.28	9.23	6	0.13	15.3	-0.026	320
		Sep. 2010	1.23 (0.74-1.73)	0.91 ± 0.33	3.69	6	0.72	20.5	-0.005	320
	Faisalabad	Sep. 2008	1.33 (0.73-1.94)	0.83 ± 0.16	6.18	6	0.40	22.2	_	320
		Mar. 2009	1.20 (0.66-1.70)	0.82 ± 0.24	4.18	6	0.65	20	-0.007	320
		Sep. 2009	0.99 (0.68-1.31)	1.11 ± 0.39	9.71	6	0.14	16.5	-0.021	320
		Mar. 2010	0.94 (0.65-1.22)	1.13 ± 0.42	9.48	6	0.15	15.7	-0.025	320
		Sep. 2010	1.23 (0.73-1.72)	0.91 ± 0.23	4.31	6	0.64	20.5	-0.006	320
	Sargodha	Sep. 2008	1.01 (0.69-1.32)	1.14 ± 0.34	6.36	6	0.38	16.8	_	320
		Mar. 2009	0.90 (0.61-1.20)	1.04 ± 0.22	3.26	6	0.78	15	-0.008	320
		Sep. 2009	0.80 (0.57-1.03)	1.18 ± 0.52	3.54	6	0.74	13.2	-0.017	320
		Mar. 2010	0.92 (0.58-1.26)	0.92 ± 0.24	6.83	6	0.34	15.3	-0.007	320
		Sep. 2010	1.10 (0.72-1.47)	1.06 ± 0.42	3.09	6	0.80	18.3	0.006	320
**Thiodicarb**	Lab-PK		0.03 (0.01-0.11)	2.28 ± 0.63	1.28	6	0.97	1	_	320
	Lahore	Sep. 2008	1.05 (0.71-1.37)	1.13 ± 0.23	8.29	6	0.22	35	_	320
		Mar. 2009	0.88 (0.61-1.15)	1.12 ± 0.32	3.99	6	0.68	29.3	-0.013	320
		Sep. 2009	0.90 (0.61-1.19)	1.07 ± 0.41	4.49	6	0.61	30	-0.011	320
		Mar. 2010	0.71 (0.51-0.89)	1.28 ± 0.25	6.13	6	0.41	23.7	-0.028	320
		Sep. 2010	0.99 (0.68-1.28)	1.17 ± 0.23	1.75	6	0.94	33	-0.004	320
	Faisalabad	Sep. 2008	1.10 (0.71-1.49)	1.01 ± 0.28	7.92	6	0.24	36.7	_	320
		Mar. 2009	0.89 (0.63-1.18)	1.09 ± 0.30	5.02	6	0.54	29.7	-0.015	320
		Sep. 2009	0.97 (0.62-1.32)	0.97 ± 0.20	4.86	6	0.56	32.3	-0.009	320
		Mar. 2010	0.76 (0.54-0.98)	1.18 ± 0.42	8.75	6	0.19	25.3	-0.027	320
		Sep. 2010	1.07 (0.71-1.43)	1.06 ± 0.23	4.11	6	0.66	35.7	-0.002	320
	Sargodha	Sep. 2008	0.85 (0.52-1.19)	0.86 ± 0.17	5.00	6	0.54	28	_	320
		Mar. 2009	0.83 (0.58-1.09)	1.14 ± 0.29	1.85	6	0.93	27.7	-0.002	320
		Sep. 2009	0.94 (0.63-1.25)	1.21 ± 0.33	4.41	6	0.62	31.2	0.007	320
		Mar. 2010	0.71 (0.53-0.90)	1.31 ± 0.44	6.35	6	0.39	23.7	-0.013	320
		Sep. 2010	1.03 (0.71-1.33)	1.17 ± 0.22	3.74	6	0.71	34	0.014	320
**Indoxacarb**	Lab-PK		0.022 (0.014-0.044)	2.19 ± 0.46	1.73	6	0.94	1	_	320
	Lahore	Sep. 2008	0.91 (0.59-1.21)	1.02 ± 0.24	3.62	6	0.73	41.4	_	320
		Mar. 2009	1.29 (0.76-1.83)	0.90 ± 0.17	2.62	6	0.86	58.6	0.025	320
		Sep. 2009	1.12 (0.69-1.54)	0.95 ± 0.25	4.76	6	0.57	50.9	0.015	320
		Mar. 2010	1.18 (0.83-1.52)	1.28 ± 0.34	10.7	6	0.10	53.6	0.019	320
		Sep. 2010	1.19 (0.78-1.59)	1.07 ± 0.24	4.58	6	0.60	54.1	0.019	320
	Faisalabad	Sep. 2008	1.21 (0.64-1.76)	0.78 ± 0.16	3.02	6	0.81	55	_	320
		Mar. 2009	1.42 (0.78-2.07)	0.85 ± 0.38	4.00	6	0.68	64.5	0.012	320
		Sep. 2009	1.87 (0.66-2.31)	0.63 ± 0.24	1.38	6	0.97	85	0.032	320
		Mar. 2010	1.17 (0.82-1.53)	1.23 ± 0.43	9.78	6	0.13	53.1	-0.002	320
		Sep. 2010	2.19 (0.37-4.00)	0.51 ± 0.11	3.19	6	0.78	99.5	0.043	320
	Sargodha	Sep. 2008	1.62 (0.56-2.69)	0.59 ± 0.18	3.77	6	0.71	73.6	_	320
		Mar. 2009	1.37 (0.73-2.00)	0.83 ± 0.42	1.98	5	0.85	62.3	-0.012	280
		Sep. 2009	1.59 (0.78-2.38)	0.80 ± 0.23	3.54	6	0.74	72.3	-0.001	320
		Mar. 2010	1.37 (0.77-1.89)	1.05 ± 0.25	12.0	6	0.07	62.2	-0.012	320
		Sep. 2010	1.96 (0.60-3.33)	0.60 ± 0.18	3.42	6	0.75	89.1	0.014	320
**Emamectin benzoate**	Lab-PK		0.09 (0.04-0.14)	2.35 ± 0.98	2.85	6	0.83	1	_	320
	Lahore	Sep. 2008	1.39 (0.91-1.86)	1.16 ± 0.26	9.23	6	0.16	15.4	_	320
		Mar. 2009	1.65 (0.59-2.70)	0.64 ± 0.22	0.41	4	0.98	18.3	0.013	240
		Sep. 2009	2.07 (0.60-3.54)	0.50 ± 0.12	7.02	6	0.32	23	0.029	320
		Mar. 2010	1.61 (0.92-2.29)	1.00 ± 0.27	9.62	6	0.14	17.9	0.011	320
		Sep. 2010	2.45 (1.35-4.26)	0.52 ± 0.26	2.66	6	0.85	27.2	0.041	320
	Faisalabad	Sep. 2008	1.35 (0.87-1.82)	1.10 ± 0.25	7.78	6	0.26	15	_	320
		Mar. 2009	1.97 (1.21-3.32)	0.61 ± 0.24	4.78	6	0.57	21.9	0.027	320
		Sep. 2009	1.47 (0.91-2.02)	1.07 ± 0.26	10.9	6	0.91	16.3	0.006	320
		Mar. 2010	2.00 (1.12-3.17)	0.76 ± 0.18	4.53	6	0.61	22.2	0.028	320
		Sep. 2010	1.89 (0.68-2.50)	0.65 ± 0.23	1.39	6	0.98	21	0.024	320
	Sargodha	Sep. 2008	1.14 (0.85-1.46)	1.31 ± 0.25	7.11	6	0.31	12.7	_	320
		Mar. 2009	1.26 (0.89-1.63)	1.33 ± 0.26	4.32	6	0.63	14	0.007	320
		Sep. 2009	1.67 (0.88-2.59)	0.73 ± 0.24	5.96	6	0.43	18.6	0.028	320
		Mar. 2010	1.70 (0.86-2.54)	0.86 ± 0.30	7.47	5	0.19	18.9	0.029	320
		Sep. 2010	1.30 (0.91-1.75)	1.10 ± 0.22	3.74	6	0.71	14.4	0.010	320
**Spinosad**	Lab-PK		0.019 (0.02-0.13)	2.71 ± 0.44	2.92	6	0.82	1	_	320
	Lahore	Sep. 2008	0.53 (0.40-0.66)	1.45 ± 0.32	3.22	6	0.78	27.9	_	320
		Mar. 2009	0.51 (0.39-0.61)	1.78 ± 0.24	3.02	6	0.81	26.8	-0.003	320
		Sep. 2009	0.48 (0.38-0.57)	1.09 ± 0.26	9.25	6	0.16	25.3	-0.007	320
		Mar. 2010	0.57 (0.44-0.69)	1.56 ± 0.42	7.74	6	0.26	30	0.005	320
		Sep. 2010	0.59 (0.43-0.72)	1.55 ± 0.42	10.2	6	0.13	31.1	0.008	320
	Faisalabad	Sep. 2008	0.63 (0.46-0.81)	1.27 ± 0.23	8.32	6	0.22	33.2	_	320
		Mar. 2009	0.73 (0.55-0.91)	1.43 ± 0.38	5.18	6	0.52	38.4	0.011	320
		Sep. 2009	0.50 (0.38-0.62)	1.52 ± 0.24	10.1	6	0.12	26.3	-0.012	320
		Mar. 2010	0.43 (0.30-0.56)	1.29 ± 0.23	12.2	6	0.06	22.6	-0.028	320
		Sep. 2010	0.95 (0.70-1.21)	1.26 ± 0.43	2.12	6	0.91	50	0.030	320
	Sargodha	Sep. 2008	0.56 (0.41-0.71)	1.33 ± 0.22	7.79	6	0.25	29.5	_	320
		Mar. 2009	0.73 (0.54-0.93)	1.29 ± 0.22	4.91	6	0.56	38.4	0.019	320
		Sep. 2009	0.52 (0.39-0.64)	1.50 ± 0.36	10.0	6	0.16	27.4	-0.005	320
		Mar. 2010	0.43 (0.29-0.56)	1.22 ± 0.23	9.93	6	0.13	22.6	-0.019	320
		Sep. 2010	0.85 (0.62-1.07)	1.44 ± 0.56	3.16	6	0.79	44.7	0.030	320

*Resistance ratio = LC_50 _field population/LC_50 _of susceptible strain

**Rate of increase or decrease in resistance *** Number of larvae tested in a bioassay

### Toxicity of insecticides to field population

The toxicity of all eleven insecticides against field population was significantly lower than Lab-PK (95% FL did not overlap, Table 1, 2).

### Organophosphates

The levels of resistance to chlorpyrifos in samples from all the three districts of Punjab were generally very high, with resistance ratios 157-266 fold. All the field populations tested with chlorpyrifos in 3 consecutive years showed very high levels of resistance (RR > 100). The highest level of resistance (266 fold) was observed in March 2009 from Faisalabad, whereas the lowest (157 fold) was from Lahore in March 2009 (Table 1). The slopes of regression lines of all the populations were significantly shallower than Lab-PK population (P < 0.05).

Among 15 populations tested for profenofos, five populations had moderate levels of resistance (24-26 fold) than the Lab-PK population, and the remaining 10 populations had high levels of resistance (34-52 fold). The highest level of resistance was found in population from Faisalabad in March 2010, whereas the lowest level was found in population from Sargodha in September 2008 (Table 1). The slopes of regression lines of all the populations were significantly shallower than the Lab-PK population (P < 0.05).

All the 15 populations tested for triazofos had high levels of resistance (41-71 fold). The highest level of resistance was seen in populations from Sargodha in September 2009, whereas the lowest level was observed in populations from Lahore in September 2009 (Table 1).

### Pyrethroids

Moderate levels of resistance was found in all the populations tested against cypermethrin (15-26 fold, Table 1) compared with the lab-PK population. The lowest level of resistance was observed in populations from Sargodha in March 2010. The slopes of regression lines of all the populations were significantly shallower than Lab-PK population (P < 0.05).

Moderate to high levels of resistance were observed in populations tested for deltamethrin (15- to 53 fold, Table 1). One population from Lahore and four populations from Sargodha had moderate levels of resistance (15-25 fold) while the remaining populations were highly resistant (31-53 fold). Of 15 populations tested against lambdacyhalothrin, only three populations from Sargodha had moderate levels of resistance with resistance ratios ranging from 21-30 fold compared with Lab-PK population (Table 1). The slopes of regression lines of all the field populations were similar (P > 0.05).

### Carbamates

Methomyl was significantly less toxic to field populations (P < 0.01) compared to Lab-PK. All the field populations tested for methomyl had moderate levels of resistance (13-22 fold) compared with Lab-PK (Table 2). The lowest level was observed in populations from Sargodha in September 2009. The slopes of regression lines of all the field populations were similar (P > 0.05) but shallower than the Lab-PK (P < 0.05).

Out of 15 populations tested for thiodicarb, three populations from Lahore, two from Faisalabad and three from Sargodha were moderately resistant with resistance ratios ranging from 24-30 fold compared with Lab-PK (Table 2). The remaining 7 populations were highly resistant to this chemical (31-37 fold).

### New insecticides

Among the 15 populations tested with indoxacarb, only one population from Faisalabad in September 2010 showed very high resistance (101 fold) while the remaining populations were highly resistant with resistance ratios ranging from 41-89 fold compared with Lab-PK (Table 2). The lowest level of resistance was found in populations from Lahore in September 2008. All the populations tested for emamectin benzoate were moderately resistant (14-27 fold) compared with Lab-PK (Table 2). The lowest level was found in populations from Sargodha in March 2009. Moderate to high levels of resistance were observed in populations tested for spinosad (23-50 fold, Table 2) compared with Lab-PK. One population from Lahore, three populations from Faisalabad and two from Sargodha had high levels of resistance while the remaining populations were moderately resistant (Table 2). The slopes of regression lines of all the field populations were similar (P > 0.05).

### Pair wise correlations between log LC_50s _of different insecticides

Correlation between emamectin benzoate and spinosad in the new chemicals group was non- significant (P > 0.05); however, resistance to emamectin benzoate was significantly (P < 0.05) correlated with profenofos and lambdacyhalothrin, but no significant correlation was found between emamectin benzoate and chlorpyrifos, triazofos, cypermethrin, deltamethrin, methomyl, thiodicarb and indoxacarb (Table 3). In contrast, spinosad had significant correlation with thiodicarb and indoxacarb but no correlation with the remaining insecticides. Indoxacarb had significant correlation with chlorpyrifos only. The LC_50 _values of the insecticides of carbamate group had highly significant (P < 0.01) correlation within the group. However, thiodicarb had also a significant correlation with deltamethrin, and methomyl with cypermethrin and deltamethrin. Within the pyrethriod group, deltamethrin and cypermethrin had significant correlation (P < 0.05). All the pyrethroids had a significant correlation with profenofos. Moreover, insecticides in organophosphate group had non significant (P > 0.05) correlation with each other (Table 3).

**Table 3:** Pairwise comparisons of correlation coefficient between log LC_50 _(µg mL^-1^) values of the tested insecticides in field populations of *Ae. albopictus*

Insecticide	Chlorpyrifos	Profenofos	Triazofos	Cypermethrin	Deltamethrin	Lambdacyhalothrin	Methomyl	Thiodicarb	Indoxacarb	Emamectin
Chlorpyrifos										
Profenofos	0.33^ns^									
Triazofos	-0.37^ns^	0.08^ns^								
Cypermethrin	0.28^ns^	0.69^<0.01^	0.29^ns^							
Deltamethrin	-0.07^ns^	0.52^<0.05^	0.37^ns^	0.52^<0.05^						
Lambdacyhalothrin	0.58^<0.05^	0.47^<0.05^	-0.36^ns^	0.37^ns^	0.39^ns^					
Methomyl	-0.02^ns^	0.04^ns^	0.27^ns^	0.47^<0.05^	0.67^<0.01^	0.38^ns^				
Thiodicarb	-0.27^ns^	-0.01^ns^	0.28^ns^	0.27^ns^	0.50^<0.05^	0.24^ns^	0.71^<0.01^			
Indoxacarb	-0.48^<0.05^	-0.08^ns^	0.54^ns^	-0.09^ns^	-0.04^ns^	-0.40^ns^	-0.06^ns^	0.30^ns^		
Emamectin benzoate	0.18^ns^	0.52^<0.05^	-0.41^ns^	0.14^ns^	0.31^ns^	0.51^<0.05^	0.08^ns^	-0.07^ns^	-0.20^ns^	
Spinosad	-0.34^ns^	-0.22^ns^	0.35^ns^	-0.07^ns^	0.16^ns^	-0.19^ns^	0.41^ns^	0.49^<0.05^	0.62^<0.05^	-0.12^ns^

(Superscripts indicate the significance of correlation)

### Stability of resistance across 3 years

From 2008 to 2010, resistance of *Ae. albopictus *to all tested insecticides remained the same. There was no indication of significant change (P > 0.05) in the rate of increase or decrease in resistance to the insecticides tested (Table 1,2).

## Discussion

Pakistan has a long history of insecticide resistance problems in cotton pests like *Helicoverpa armigera, Bemisia tabaci*, *Aphis gosypii *[25], *Spodoptera litura *[24] and even in the generalist predator, the green lacewing, *Chrysoperla carnea *[16]. Here we have shown a strong indication of resistance in *Ae. albopictus *collected from areas of high agrochemical use compared to the Lab-PK population with zero agrochemicals exposure. The present experiments were carried out to evaluate the resistance of three insecticides from each of organophosphate, pyrethroid, new chemicals and two from carbamates. These insecticides have intensively been used to combat various cotton pests in Pakistan for the last two decades [26]. The experiments were conducted for 3 consecutive years (2008-2010), and the results of bioassays showed varying degrees of resistance in field populations. Resistance in *Ae. albopictus *to chlorpyrifos was generally very high while moderate to high levels of resistance were found with remaining insecticides. Insect populations should be considered susceptible if a resistance ratio of 10 is exhibited [27], however, none of the field populations was found to have resistance ratio 10 or below 10. The present studies suggest that *Ae. albopictus *might have evolved resistance to agrochemicals due to possible cross resistance mechanisms among various agrochemicals.

Pair wise correlation coefficient comparisons of log LC_50 _values of insecticides for field populations showed positive correlations among most of the insecticides (Table 3), suggesting a cross resistance mechanism. The presence of two divergent patterns of correlation within agrochemicals indicates that more than one mechanism of resistance exists for imparting resistance to agrochemicals in *Ae. albopictus*. High levels of resistance to most of the insecticides might be due to multiple resistance mechanism [17]. The mixing of new chemicals with conventional insecticides could be responsible for creating multiple resistance problems, which has been reported in *Spodoptera *sp. from other parts of the world [28]. Owing to the common practice of mixing new compounds with conventional insecticides to control cotton pests in Pakistan [17] it would be untimely to conclude that cross resistance exists in *Ae. albopictus *against these agrochemicals. However further studies are required to confirm whether the cross resistance between insecticides exists by selecting *Ae. albopictus *population in the laboratory with representative insecticides.

In the present investigations *Ae. albopictus *larvae were found resistant to all classes of tested chemicals which could be due to one or more than one resistance mechanisms involved. The resistance of *Aedes *larvae to pyrethroids and organophosphates has also been reported from other parts of the world [1]. Insecticide resistance mechanism in mosquitoes has extensively been studied in the past [29]. The resistance to pyrethroids in mosquitoes is mainly conferred by two mechanisms: (a) mutation in the voltage-gated sodium channel or (b) by elevated levels of monooxygenases [30,31]. In contrast, over expression of esterases by gene amplification or mutation provides considerable organophosphate (and to some extent carbamate) resistance in mosquitoes, and has been considered an evolutionary response to selection by organophosphates and carbamates [31]. Monooxygenases play a minor role in organophosphate resistance, and little, if any, in resistance to carbamates [32].

In the current study, rate of increase or decrease of resistance to insecticides in the field population of *Ae. albopictus *was minimal, suggesting that resistance was stable in the populations collected from various locations. The stability of resistance in field collected samples conferred at least one justification. The resistance might have been near fixation, leading to a very slow increase in heterozygosity owing to combination of ecological, biological and/or biochemical (reduced detoxification capacity) factors [16,33].

Though the *Aedes *larvae have not been directly exposed to agrochemicals applied for the cotton pest management, the results of present study clearly showed the field evolved resistance to agrochemicals. The use of insecticides in agricultural crop protection could indeed affects resistance development in disease vectors [13,34,35]. The cotton crop in Pakistan is usually sprayed with more than 34 insecticides of different chemical classes, including premix and tank mix products [26]. These chemicals are mainly organophosphates, carbamates, pyrethroids, neonicotinoids and new chemistries and are used either as a single formulation or in combinations of two or three insecticides of different classes, the final aim being to generate a synergistic effect of insecticides for a better pest management. After pesticide treatments in agricultural crops, insecticides residues drift into mosquito breeding sites [9]. These residues have lethal effects on larvae of some populations of mosquitoes whereas they exercise a selective pressure on other populations, leading to the emergence of resistant populations [10]. Moreover, considerable agrochemical-insect contact could occur during mosquito flights between breeding habitats and blood sources and resting places, potentially increasing selection pressure to insecticides [9]. Moreover insecticides used in public health programs against disease vectors are similar to those used for years in agriculture [10]. Several hypotheses concerning the resistance in disease vectors have emerged e.g. some researchers incriminate pesticide use in cotton and rice fields as the main source of resistance selection in several species of mosquitoes in rural environments [35-39]. One important threat that may compromise future control efforts is the potential for cross resistance between organophosphates and pyrethroids. Resistance to pyrethroids has generally been associated with cross resistance to DDT [40], however, an esterase-based resistance mechanism in *An. albimanus *conferred cross resistance between pyrethroid (deltamethrin) and organophosphate (fenithrothion) [41]. In areas where organophosphate has extensively been used for agricultural pest control, such cross resistance may pose a potential threat to future control of the dengue vector.

## Conclusions

We now plan to look for pesticide residues in mosquito breeding sites to confirm the actual involvement of agrochemicals in the selection of resistance in *Ae. albopictus*. In conclusion, it is recommended that regular resistance surveillance should first be focused in areas where dengue fever transmission and intensive chemical agricultural pest control coincide, because these areas are more prone to develop insecticide resistance in *Ae. albopictus*. Continuous resistance monitoring will also result to identify the efficacy of compounds for dengue control and to facilitate selection of compounds with the greatest promise to minimize dengue infections. Moreover, public awareness about dengue, cooperation with public health campaigns to eliminate larval Aedes breeding habitats and Insecticide Resistance Management in combination with Integrated Pest and Vector Management are recommended strategies for controlling dengue vectors and to reduce risks to humans as well as environmental health.

## Competing interests

The authors declare that they have no competing interests.

## Authors' contributions

HAAK conceived, designed, performed the study, analyzed the data and wrote the manuscript. WA supervised the study and helped draft the manuscript. KS helped in mosquito collections and bioassays and EASS helped draft the manuscript. All authors approved the final version of the manuscript.
